# Self-medication and pain in the European Union: Gender differences and associated factors

**DOI:** 10.1016/j.pmedr.2026.103488

**Published:** 2026-05-04

**Authors:** Spencer Yeamans, Ángel Gil-De-Miguel, Valentín Hernández-Barrera, Pilar Carrasco-Garrido

**Affiliations:** Department of Medical Specialties and Public Health. Preventative Medicine and Public Health area, Universidad Rey Juan Carlos. Av. de Atenas, s/n, 28922 Alcorcón, Madrid, Spain

**Keywords:** Over-the-counter, OTC, Non-prescription, Gender, Sex, European health interview survey, EHIS

## Abstract

**Objective:**

Pain management, including self-medication, is an increasingly critical responsibility of public health. This study examines the prevalence and associated factors of self-medication in individuals experiencing pain in the European Union.

**Methods:**

This study performs multivariable analysis utilizing data from wave three (2018–2020) of the European Health Interview Survey (*N* = 149,349).

**Results:**

Overall self-medication prevalence is 42.7%, with rates ranging from 19.0% to 85.1%.Self-medication prevalence is affected by pain severity and the association differs by sex: men are most likely to self-medicate at moderate pain levels (adjusted odds ratio [AOR] = 1.25;95%CI = 1.16,1.35), whereas women are most likely to self-medicate at severe/very severe pain levels (AOR = 1.30;95%CI = 1.20,1.40). Additionally, an association between depression and self-medication is found exclusively in men (AOR = 1.38;95%CI = 1.19,1.59). Several factors are also associated with self-medication in both sexes, notably improved education (male AOR = 2.70;95%CI = 2.11,3.45/female AOR = 4.09;95%CI = 3.44,4.87) and over-the-counter medication availability outside pharmacies (male AOR = 1.72;95%CI = 1.61,1.84/female AOR = 2.25;95%CI = 2.13,2.37). Finally, we find that economic barriers to healthcare access influence self-medication (male AOR = 1.31;95%CI = 1.11,1.55/female AOR = 1.33;95%CI = 1.18,1.49).

**Conclusions:**

The findings highlight sex differences and the impact of pain severity on self-medication. The results also demonstrate cross-country variation in both self-medication practices and pain, underscoring the need for better education about responsible self-medication, a review of over-the-counter medication policies, and reduced healthcare barriers.

## Highlights

• Self-medication is more prevalent among individuals in pain than general population.
• Major sex differences exist for self-medication in individuals experiencing pain.
• Education is one of the strongest influences on self-medication behavior.
• Over-the-counter medication availability outside of pharmacies increases their use.

## 1 Introduction

Pain, an unpleasant sensory and emotional experience associated with actual or potential tissue damage (Raja et al., 2020), is a characteristic of a multitude of the most substantial leaders in disability-adjusted life-years andyearslivedwithdisabilityintheglobalburdenofdisease(GBD) (Vos et al., 2020). Pain may be chronic or acute, with prevalence estimates varying significantly, including across countries, with a 52-country sample finding a range of 9.9%–50.3% during a 30-day window, wherein European countries reported the highest rates (Zimmer et al., 2022).

One common method for reducing and preventing pain is self-medication, or the use of over-the-counter (OTC) and prescription medications, herbal products, home-made remedies, nutritional supplements and vitamins without strict adherence to authorized health care professionals' instruction regarding indication, dose, and duration of treatment (Baracaldo-Santamaría et al., 2022). The prevalence of self-medication in the European Union general population during a 14-day window has been previously established at 34.3%, and self-medication is associated with a multitude of demographic, socioeconomic, lifestyle, and health factors (Yeamans et al., 2024). The most common self-medication treatment is the use of analgesics (Perrot et al., 2019; Sarganas et al., 2015). OTC analgesic use is growing worldwide, in all kinds of populations, independent of drug accessibility or country (Perrot et al., 2019), while prescription analgesic use has remained constant (Sarganas et al., 2015). Simultaneously, concerns regarding overconsumption and the dangers posed by non-prescription analgesics are rising, with calls for greater information among the public about the risks associated with common painkillers like paracetamol (Petrides et al., 2023). Mehuys et al. found the clinical picture of people who self-medicate with OTC analgesics “looked worse than expected” (Mehuys et al., 2019).

Self-medication for those dealing with pain is not limited, however, to analgesics. Pain exists comorbidly with many conditions which are self-medicated with substances other than analgesics, including cardiovascular agents, dietary complements and alternative medicine components (Jerez-Roig et al., 2014). In addition, heartburn/acid reflux, allergies/hay fever, chronic sleeping problems, irritable bowel syndrome, ulcers, or urinary/bladder problems may also cause pain and necessitate self-medication beyond analgesics (Halvorsen et al., 2016).

Given the growing share of non-communicable disease and injury in the GBD due to an aging population, improvements in health infrastructure, and lifestyle changes (Halvorsen et al., 2016; Vos et al., 2020), particularly within the European Union, managing patient pain is an increasingly central responsibility of public health. Although prior research using European Health Interview Survey (EHIS) data has examined self-medication in the general population of the European Union, evidence remains limited with respect to individuals experiencing pain, an epidemiologically distinct subgroup for whom medication use differs substantially. As a result, this study seeks to fill that gap by establishing self-medication prevalence and associated factors for individuals with varying levels of pain in the European Union, as well as assess the relationship between their pain severity and self-medication behavior, with data from the third wave (2018–2020) of the EHIS.

## 2 Methods

### 2.1 Data source and study population

This observational study utilizes anonymized data from wave three of the cross-sectional EHIS (Eurostat (European Commission), 2020). Surveys were conducted between January 2018 and September 2020 on a broad representative sample of the population, consisting of the non-institutionalized subjects aged 15 and over residing in European Union countries. Twenty-six of the twenty-seven European Union member states are represented in this study, as French data was unavailable via Eurostat. Data from Malta for the nationality variable was also excluded due to anonymization practices. Results are comparable between countries due to the use of a common regulatory framework. The total weighted sample size for this study is 149,349. The microdata was anonymized by the Eurostat office in accordance with the European Union regulation 2018/255 on the European Health Interview Survey. The Universidad Rey Juan Carlos Ethics Committee confirmed the lack of need for ethics committee approval for this project.

### 2.2 Study measures

The dependent variable is self-medication, measured dichotomously (yes/no) via the question “During the past two weeks, have you used any medicines or herbal medicines or vitamins not prescribed by a doctor?” excluding contraceptive pills or hormones used solely for contraception. The study sample is divided into three groups in function to their response to the question “How much bodily pain have you had during the past 4 weeks?” Responses were grouped into the categories very mild/mild, moderate, and severe/very severe, where very mild/mild was used as the reference group. Respondents were instructed to answer according to whatever medication they were taking. Non-responses and proxy responses to both questions were removed to improve data homogenization.

Independent variables in this study include demographic, socioeconomic, lifestyle, and health-related characteristics. Demographic variables consist of age, sex, and nationality (native-born, born in another European Union member state, born in a non-European Union country). Socioeconomic variables include education (no formal education, primary school, secondary school, higher education) and employment status (employed, unemployed, or inactive), where inactive includes retirees, students, and those performing domestic tasks, carrying out compulsory service, or unable to work for health reasons. Lifestyle variables include smoking (yes/no), alcohol consumption (more or less than once a month), and physical activity levels (low, moderate, high). Construction of the physical activity independent variable was guided by Jemna et al., 2022, combining the methodology of the International Physical Activity Questionnaire with World Health Organization recommendations on physical activity and using information on the type (sports, strength training, walking, cycling), frequency, and duration of physical activity according to age groups.

To understand the impact of the availability of OTC medications on self-medication, clusters developed by Oleszkiewicz et al., 2021 were utilized. Three categories were formed. The first category, “Pharmacy-only OTC availability” refers to countries where OTC medicines are available exclusively in pharmacies (Austria/Belgium/Cyprus/Estonia/Finland/France/Greece/Lithuania/Luxembourg/Latvia/Malta/Slovakia/Spain). The “OTC availability outside of pharmacies” category contains countries where OTC medicines on general sales lists may be purchased from sales points other than pharmacies (Poland/Ireland/the Netherlands/Slovenia/Hungary/Italy/Czechia/Denmark/Sweden). The final category “OTC availability in limited-service pharmacies” includes countries where OTC medications are available in pharmacies where it is not possible to consult with qualified staff when making a purchase (Bulgaria/Croatia/Germany/Portugal/Romania/Switzerland). Additional health variables include presence of a long-standing health problem (yes/no), self-perceived health (very good/good, fair, bad/very bad), and self-reported depression (yes/no). Visit to a general practitioner/family doctor and visit to a medical/surgical specialist in the past 12 months are also measured (yes/no). Finally, an evaluation of unmet health care needs in the past 12 months due to inability to afford medical examination/treatment was included (yes, no, no need for health care).

### 2.3 Statistical analysis

We calculated the prevalence of self-medication in individuals experiencing varying levels of pain in the past four weeks by country and for each independent variable, selected based on relevancy in the literature, via answers to the dependent variable. For each country, odds ratios (OR) with 95% confidence intervals (95% CI) were estimated, stratified by sex. With respect to the independent variables, to measure the difference in self-medication between varying pain severities, we utilized logistic regression. Furthermore, to estimate the independent effect of the variables on self-medication, including pain severity, we calculated the adjusted odds ratios (AOR) and the 95%CIs via multivariable logistic regression analysis utilizing the methodology used by Hosmer et al. (Hosmer Jr et al., 2013). All analysis was stratified by sex. Estimates were made using the survey command (svy function) in Stata®, enabling the incorporation of sample design and weights in all statistical calculations. Statistical significance is set as 2-tailed α < 0.05. The figure was created in R using the forestploter package.

## 3 Results

### 3.1 Descriptive statistics and prevalences

The characteristics of the study population are displayed in Table 1**.** Among individuals experiencing during the four-week reference period, self-medication in the preceding two weeks was reported by 42.7% of respondents, with higher prevalence among women than men (47.00% vs. 36.9%). Self-medication prevalence is highest with moderate pain (male = 39.4%, female = 47.8%), followed by severe/very severe pain (male = 38.4%, female = 47.4%) and very mild/mild pain (male = 35.5%, female = 46.4%). Additionally, The prevalence of self-medication by pain severity and country is described in **Supplementary Table 1.** Self-medication prevalence ranged from 19.0% in Romanian men (very mild/mild pain) to 85.1% in Finnish women (moderate pain). Spain also has low self-medication prevalence, with no sex or pain subgroup surpassing 25% prevalence.

**Table 1:** Description of non-institutionalized residents aged 15 and over experiencing pain in the European Union. European Health Interview Survey Wave 3 (2018–2020).

Male *N* = 61,047 (42.7%)				Female *N* = 88,302 (57.3%)		Both Sexes *N* = 149,349 (100%)	
Variable	Category	N	% (95%CI)	N	% (95%CI)	N	% (95%CI)
Self-medication in the past two weeks	Yes	22,064	36.9 (36.9, 37.0)	41,469	47.0 (47.0, 47.0)	63,533	42.7 (42.7, 42.7)
	No	38,983	63.1 (63.1, 63.1)	46,833	53.0 (53.0, 53.0)	85,816	57.3 (57.3, 57.3)
Intensity of pain in the past four weeks	Very mild/mild	35,194	59.2 (59.1, 59.2)	43,480	50.9 (50.9, 50.9)	78,674	54.4 (54.4, 54.4)
	Moderate	17,023	26.7 (26.7, 26.7)	27,526	30.4 (30.3, 30.4)	44,549	28.8 (28.8, 28.8)
	Severe/very severe	8830	14.1 (14.1, 14.2)	17,296	18.7 (18.7, 18.8)	26,126	16.8 (16.8, 16.8)
Age	15–24 years	3697	8.2 (8.2, 8.2)	5006	7.4 (7.4, 7.4)	8703	7.7 (7.7, 7.7)
	25–44 years	12,560	25.3 (25.3, 25.4)	17,113	23.4 (23.3, 23.4)	29,673	24.2 (24.2, 24.2)
	45–64 years	22,956	38.3 (38.2, 38.3)	31,361	36.0 (35.9, 36.0)	54,317	36.9 (36.9, 36.9)
	65–74 years	12,145	15.5 (15.5, 15.5)	17,189	16.4 (16.4, 16.4)	29,334	16.0 (16.0, 16.0)
	75+ years	9689	12.7 (12.7, 12.7)	17,633	16.9 (16.9, 16.9)	27,322	15.1 (15.1, 15.1)
Nationality*	Native-born	55,258	91.0 (91.0, 91.0)	79,733	90.9 (90.9, 90.9)	134,991	90.9 (90.9, 90.9)
	Born in another EU state	2220	2.9 (2.9, 2.9)	3368	3.1 (3.1, 3.1)	5588	3.0 (3.0, 3.0)
	Born in non-EU country	3456	6.1 (6.1, 6.1)	5017	6.0 (6.0, 6.0)	8473	6.1 (6.1, 6.1)
Education level	No formal education	1164	1.7 (1.7, 1.7)	3067	2.7 (2.7, 2.7)	4231	2.3 (2.3, 2.3)
	Primary school	17,071	23.5 (23.5, 23.5)	27,987	28.1 (28.1, 28.1)	45,058	26.1 (26.1, 26.1)
	Secondary school	25,864	49.0 (49.0, 49.0)	33,181	44.4 (44.4, 44.4)	59,045	46.4 (46.4, 46.4)
	Higher education	16,482	25.9 (25.9, 25.9)	23,484	24.8 (24.8, 24.8)	39,966	25.2 (25.2, 25.2)
Employment status	Employed	28,837	52.9 (52.9, 52.9)	33,196	42.3 (42.3, 42.3)	62,033	46.8 (46.8, 46.9)
	Unemployed	2600	4.3 (4.3, 4.3)	3529	4.0 (4.0, 4.0)	6129	4.2 (4.2, 4.2)
	Inactive	29,218	42.8 (42.7, 42.8)	51,053	53.7 (53.6, 53.7)	80,271	49.0 (49.0, 49.0)
Smoking	Yes	15,887	28.6 (28.6, 28.6)	14,662	18.7 (18.7, 18.8)	30,549	23.0 (23.0, 23.0)
	No	44,539	71.4 (71.4, 71.4)	72,725	81.3 (81.3, 81.3)	117,264	77.0 (77.0, 77.1)
Alcohol consumption	More than once a month	25,115	50.1 (50.1, 50.1)	17,037	24.9 (24.9, 24.9)	42,152	35.6 (35.6, 35.6)
	Once a month or less	26,084	49.9 (49.9, 49.9)	56,929	75.1 (75.1, 75.2)	83,013	64.4 (64.4, 64.4)
Physical activity	Low	33,823	52.4 (52.4, 52.4)	53,076	56.4 (56.4, 56.4)	86,899	54.7 (54.7, 54.7)
	Moderate	11,843	18.8 (18.8, 18.8)	16,960	20.5 (20.5, 20.6)	28,803	19.8 (19.8, 19.8)
	High	15,103	28.8 (28.8, 28.8)	17,789	23.1 (23.1, 23.1)	32,892	25.5 (25.5, 25.5)
Medication availability cluster	Pharmacy-only OTC availability	17,354	22.1 (22.1, 22.1)	26,748	23.6 (23.6, 23.6)	44,102	22.9 (22.9, 23.0)
	OTC availability outside of pharmacies	27,885	40.4 (40.4, 40.4)	39,161	39.6 (39.6, 39.6)	67,046	40.0 (39.9, 40.0)
	OTC availability in limited-service pharmacies	15,808	37.5 (37.5, 37.5)	22,393	36.8 (36.8, 36.8)	38,201	37.1 (37.1, 37.1)
Self-perceived health	Very good/good	31,963	56.3 (56.2, 56.3)	42,156	52.2 (52.2, 52.2)	74,119	53.9 (53.9, 53.9)
	Fair	20,606	31.4 (31.4, 31.4)	32,288	34.3 (34.3, 34.3)	52,894	33.0 (33.0, 33.1)
	Bad/very bad	8218	12.4 (12.4, 12.4)	13,528	13.6 (13.5, 13.6)	21,746	13.1 (13.1, 13.1)
Long-standing health problem	Yes	36,998	60.0 (59.9, 60.0)	57,836	65.4 (65.4, 65.4)	94,834	63.1 (63.1, 63.1)
	No	23,654	40.1 (40.0, 40.1)	29,940	34.6 (34.6, 34.6)	53,594	36.9 (36.9, 37.0)
Depression**	Yes	4738	8.6 (8.5, 8.6)	10,859	12.1 (12.1, 12.1)	15,597	10.6 (10.6, 10.6)
	No	55,712	91.5 (91.4, 91.5)	76,571	87.9 (87.9, 87.9)	132,283	89.4 (89.4, 89.4)
Visit to a general practitioner or family doctor**	Yes	48,502	79.8 (79.8, 79.8)	75,590	86.0 (86.0, 86.0)	124,092	83.3 (83.3, 83.3)
	No	12,360	20.2 (20.2, 20.3)	12,447	14.0 (14.0, 14.1)	24,807	16.7 (16.7, 16.7)
Visit to a medical or surgical specialist**	Yes	33,793	55.9 (55.9, 55.9)	56,134	65.4 (65.4, 65.4)	89,927	61.3 (61.3, 61.4)
	No	26,836	44.1 (44.1, 44.1)	31,560	34.6 (34.6, 34.6)	58,396	38.7 (38.7, 38.7)
Unmet need for health care due to inability to afford medical examination or treatment**	Yes	3316	4.8 (4.8, 4.8)	6355	6.4 (6.4, 6.4)	9671	5.7 (5.7, 5.7)
	No	42,276	73.6 (73.6, 73.6)	63,262	75.8 (75.7, 75.8)	105,538	74.8 (74.8, 74.8)
	No need for health care	12,835	21.6 (21.6, 21.6)	15,222	17.9 (17.9, 17.9)	28,057	19.4 (19.4, 19.5)

* = does not include data from Malta; ** = in the past 12 months; 95%CI = 95% confidence intervals; EU = European Union; Inactive = retirees, students, and those performing domestic tasks, carrying out compulsory service, or unable to work for health reasons.

Furthermore, the prevalence of self-medication among males and females for each independent variable is presented in Table 2 and Table 3, respectively. Self-medication is most common among adults aged 25–44 across all pain levels (male:40.6%/46.5%/45.2%, female:52.2%/55.3%/57.8%). The lowest prevalence occurs in those aged 75 or older (male:29.4%/34.9%/30.2%, female:36.3%/38.8%/39.6%). Overall prevalence values for each independent variable are provided in **Supplementary Table 2**.

**Table 2:** Prevalence of self-medication in non-institutionalized male residents aged 15 and over experiencing pain in the European Union. European Health Interview Survey Wave 3 (2018–2020).

Variable	Category	Very Mild/Mild Pain		Moderate Pain		Severe/Very Severe Pain	
		N	% (95%CI)	N	% (95%CI)	N	% (95%CI)
Age	15–24 years	980	38.6 (38.6, 38.7)	292	39.7 (39.6, 39.8)	121	42.4 (42.2, 42.5)
	25–44 years	3269	40.6 (40.6, 40.6)	1309	46.5 (46.4, 46.5)	601	45.2 (45.1, 45.3)
	45–64 years	4445	33.7 (33.7, 33.7)	2400	37.9 (37.9, 37.9)	1386	38.4 (38.4, 38.5)
	65–74 years	2259	32.5 (32.4, 32.5)	1320	37.5 (37.5, 37.6)	637	37.3 (37.3, 37.4)
	75+ years	1373	29.4 (29.3, 29.4)	1076	34.9 (34.9, 35.0)	596	30.2 (30.2, 30.3)
Nationality*	Native-born	11,134	35.5 (35.4, 35.5)	5809	39.3 (39.2, 39.3)	2993	38.4 (38.3, 38.4)
	Born in another EU state	501	40.6 (40.5, 40.7)	214	34.3 (34.2, 34.5)	103	32.7 (32.6, 32.9)
	Born in non-EU country	678	33.9 (33.8, 33.9)	363	42.9 (42.8, 43.0)	237	40.3 (40.2, 40.4)
Education level	No formal education	86	14.9 (14.8, 15.0)	78	19.0 (18.8, 19.1)	69	25.4 (25.2, 25.6)
	Primary school	2286	27.2 (27.2, 27.2)	1485	28.7 (28.7, 28.8)	902	30.4 (30.4, 30.5)
	Secondary school	5389	36.5 (36.5, 36.5)	3047	42.9 (42.9, 43.0)	1539	40.9 (40.8, 40.9)
	Higher education	4476	41.0 (40.9, 41.0)	1752	46.1 (46.0, 46.1)	809	44.6 (44.5, 44.7)
Employment status	Employed	6933	37.7 (37.6, 37.7)	2933	42.7 (42.7, 42.8)	1336	42.7 (42.6, 42.7)
	Unemployed	400	31.8 (31.7, 31.9)	247	31.3 (31.2, 31.4)	175	38.4 (38.3, 38.5)
	Inactive	4906	32.5 (32.5, 32.6)	3175	36.8 (36.7, 36.8)	1813	35.1 (35.0, 35.1)
Smoking	Yes	3053	34.3 (34.3, 34.3)	1638	39.1 (39.0, 39.1)	995	39.5 (39.4, 39.5)
	No	9186	36.0 (36.0, 36.1)	4713	39.5 (39.5, 39.6)	2320	37.9 (37.8, 37.9)
Alcohol consumption	More than once a month	5600	36.2 (36.2, 36.2)	2528	41.6 (41.6, 41.7)	1218	40.3 (40.2, 40.3)
	Once a month or less	5241	34.3 (34.3, 34.3)	2948	38.4 (38.3, 38.4)	1653	38.2 (38.1, 38.2)
Physical activity	Low	5942	33.0 (33.0, 33.0)	3496	36.5 (36.5, 36.5)	2008	36.2 (36.1, 36.2)
	Moderate	2538	35.0 (35.0, 35.1)	1190	40.4 (40.3, 40.4)	530	37.6 (37.5, 37.7)
	High	3820	40.0 (40.0, 40.0)	1689	44.9 (44.8, 44.9)	793	44.0 (44.0, 44.1)
Medication availability cluster	Pharmacy-only OTC availability	3890	34.0 (34.0, 34.0)	1996	34.9 (34.9, 35.0)	1136	36.8 (36.8, 36.9)
	OTC availability outside of pharmacies	5609	39.8 (39.7, 39.8)	3026	43.7 (43.6, 43.7)	1475	42.1 (42.0, 42.1)
	OTC availability in limited-service pharmacies	2827	32.0 (32.0, 32.0)	1375	37.2 (37.2, 37.3)	730	35.4 (35.4, 35.5)
Self-perceived health	Very good/good	8178	36.2 (36.2, 36.3)	2524	41.5 (41.5, 41.5)	897	40.2 (40.1, 40.3)
	Fair	3506	34.6 (34.6, 34.7)	2800	38.3 (38.3, 38.3)	1134	38.8 (38.8, 38.9)
	Bad/very bad	600	29.9 (29.9, 30.0)	1052	36.6 (36.6, 36.7)	1301	36.7 (36.6, 36.7)
Long-standing health problem	Yes	6603	36.3 (36.3, 36.3)	4616	39.2 (39.2, 39.2)	2734	38.4 (38.4, 38.4)
	No	5658	34.8 (34.8, 34.8)	1741	39.7 (39.7, 39.8)	593	38.3 (38.2, 38.4)
Depression**	Yes	714	42.7 (42.7, 42.8)	613	42.5 (42.4, 42.6)	604	42.4 (42.3, 42.5)
	No	11,466	35.0 (35.0, 35.0)	5673	38.8 (38.8, 38.8)	2699	37.4 (37.3, 37.4)
Visit to a general practitioner or family doctor**	Yes	9357	36.0 (36.0, 36.0)	5411	38.9 (38.9, 38.9)	2999	37.8 (37.7, 37.8)
	No	2944	34.3 (34.3, 34.4)	973	42.3 (42.2, 42.3)	335	44.8 (44.7, 44.9)
Visit to a medical or surgical specialist**	Yes	6217	36.7 (36.7, 36.8)	4022	39.7 (39.6, 39.7)	2412	38.2 (38.1, 38.2)
	No	6064	34.5 (34.5, 34.5)	2346	39.0 (39.0, 39.1)	916	39.1 (39.0, 39.1)
Unmet need for health care due to inability to afford medical examination or treatment**	Yes	475	41.7 (41.6, 41.8)	446	43.0 (42.9, 43.1)	414	46.5 (46.4, 46.6)
	No	8466	35.5 (35.5, 35.6)	4627	39.1 (39.1, 39.1)	2430	37.9 (37.8, 37.9)
	No need for health care	2963	35.6 (35.6, 35.6)	1065	40.4 (40.3, 40.4)	366	37.4 (37.3, 37.5)

* = does not include data from Malta; ** = in the past 12 months; 95%CI = 95% confidence intervals; EU = European Union; Inactive = retirees, students, and those performing domestic tasks, carrying out compulsory service, or unable to work for health reasons.

**Table 3:** Prevalence of self-medication in non-institutionalized female residents aged 15 and over experiencing pain in the European Union. European Health Interview Survey Wave 3 (2018–2020).

Very Mild/Mild Pain					Moderate Pain	Severe/Very Severe Pain	
Variable	Category	N	% (95%CI)	N	% (95%CI)	N	% (95%CI)
Age	15–24 years	1565	45.8 (45.8, 45.9)	577	51.3 (51.2, 51.4)	304	50.2 (50.0, 50.3)
	25–44 years	5264	52.2 (52.2, 52.3)	2427	55.3 (55.3, 55.4)	1412	57.8 (57.7, 57.8)
	45–64 years	7556	46.9 (46.9, 46.9)	4936	50.1 (50.1, 50.1)	2927	48.7 (48.7, 48.8)
	65–74 years	3571	42.9 (42.9, 42.9)	2640	43.6 (43.5, 43.6)	1509	44.6 (44.5, 44.7)
	75+ years	2400	36.3 (36.3, 36.3)	2459	38.8 (38.8, 38.9)	1922	39.6 (39.5, 39.6)
Nationality*	Native-born	18,364	46.6 (46.6, 46.6)	11,785	48.0 (48.0, 48.0)	7195	47.6 (47.6, 47.7)
	Born in another EU state	837	48.5 (48.5, 48.6)	508	51.6 (51.4, 51.7)	339	51.6 (51.4, 51.7)
	Born in non-EU country	1091	41.4 (41.3, 41.4)	727	42.3 (42.2, 42.4)	520	42.0 (41.9, 42.1)
Education level	No formal education	164	13.8 (13.8, 13.9)	222	16.9 (16.8, 17.0)	228	18.7 (18.6, 18.8)
	Primary school	4190	35.3 (35.3, 35.4)	3348	36.9 (36.9, 36.9)	2494	37.5 (37.5, 37.6)
	Secondary school	8293	48.1 (48.1, 48.2)	5560	52.7 (52.7, 52.7)	3411	54.1 (54.0, 54.1)
	Higher education	7571	55.1 (55.0, 55.1)	3844	56.8 (56.8, 56.9)	1896	58.8 (58.8, 58.9)
Employment status	Employed	9940	51.0 (51.0, 51.0)	5124	55.0 (55.0, 55.0)	2609	54.8 (54.8, 54.9)
	Unemployed	677	36.9 (36.8, 37.0)	459	39.0 (38.9, 39.1)	367	40.9 (40.8, 41.0)
	Inactive	9632	42.5 (42.5, 42.6)	7389	43.5 (43.5, 43.5)	5049	44.2 (44.2, 44.3)
Smoking	Yes	3333	47.0 (47.0, 47.0)	2272	50.7 (50.7, 50.7)	1517	49.4 (49.3, 49.4)
	No	16,912	46.4 (46.4, 46.4)	10,666	47.2 (47.2, 47.3)	6499	47.1 (47.1, 47.1)
Alcohol consumption	More than once a month	5016	50.1 (50.1, 50.2)	2561	53.0 (53.0, 53.1)	1331	53.9 (53.8, 53.9)
	Once a month or less	13,102	45.0 (45.0, 45.1)	8716	47.2 (47.2, 47.3)	5672	47.4 (47.3, 47.4)
Physical activity	Low	10,356	43.1 (43.1, 43.1)	7395	43.3 (43.2, 43.3)	5107	43.9 (43.9, 44.0)
	Moderate	4633	48.0 (48.0, 48.1)	2592	51.1 (51.0, 51.1)	1331	51.3 (51.2, 51.4)
	High	5306	51.9 (51.9, 52.0)	3002	57.1 (57.1, 57.2)	1607	55.7 (55.7, 55.8)
Medication availability cluster	Pharmacy-only OTC availability	6705	40.5 (40.5, 40.6)	4348	40.4 (40.4, 40.4)	2832	40.9 (40.8, 40.9)
	OTC availability outside of pharmacies	8936	52.4 (52.4, 52.4)	5880	53.6 (53.5, 53.6)	3551	54.3 (54.3, 54.4)
	OTC availability in limited-service pharmacies	4715	43.7 (43.7, 43.7)	2811	46.3 (46.2, 46.3)	1691	44.2 (44.1, 44.2)
Self-perceived health	Very good/good	13,400	47.4 (47.3, 47.4)	5048	50.8 (50.8, 50.9)	2055	51.3 (51.3, 51.4)
	Fair	5996	44.7 (44.6, 44.7)	6020	47.0 (47.0, 47.0)	3002	48.6 (48.6, 48.6)
	Bad/very bad	915	41.5 (41.4, 41.5)	1924	41.9 (41.9, 42.0)	2989	43.7 (43.7, 43.7)
Long-standing health problem	Yes	10,999	46.3 (46.3, 46.3)	9768	47.3 (47.3, 47.3)	6754	47.4 (47.4, 47.4)
	No	9266	46.5 (46.4, 46.5)	3191	49.1 (49.1, 49.1)	1280	47.7 (47.6, 47.7)
Depression**	Yes	1525	49.1 (49.0, 49.2)	1756	48.8 (48.7, 48.8)	1765	45.7 (45.7, 45.8)
	No	18,586	46.1 (46.1, 46.1)	11,090	47.5 (47.5, 47.6)	6217	47.8 (47.7, 47.8)
Visit to a general practitioner or family doctor**	Yes	16,546	46.4 (46.4, 46.5)	11,519	47.5 (47.5, 47.5)	7403	47.3 (47.3, 47.3)
	No	3774	46.2 (46.2, 46.3)	1498	50.9 (50.9, 51.0)	648	48.9 (48.8, 49.0)
Visit to a medical or surgical specialist**	Yes	11,949	48.0 (48.0, 48.0)	8848	48.6 (48.6, 48.7)	6161	48.3 (48.2, 48.3)
	No	8330	44.2 (44.2, 44.2)	4143	46.1 (46.1, 46.2)	1876	44.9 (44.9, 45.0)
Unmet need for health care due to inability to afford medical examination or treatment**	Yes	1048	54.5 (54.4, 54.6)	1106	50.4 (50.3, 50.5)	1070	52.6 (52.5, 52.7)
	No	14,452	46.5 (46.5, 46.5)	9598	47.4 (47.4, 47.4)	5858	46.8 (46.7, 46.8)
	No need for health care	4209	46.1 (46.1, 46.1)	1897	50.6 (50.5, 50.7)	846	49.3 (49.2, 49.4)

* = does not include data from Malta; ** = in the past 12 months; 95%CI = 95% confidence intervals; EU = European Union; Inactive = retirees, students, and those performing domestic tasks, carrying out compulsory service, or unable to work for health reasons.

### 3.2 Multivariable analysis

**Fig. 1:**
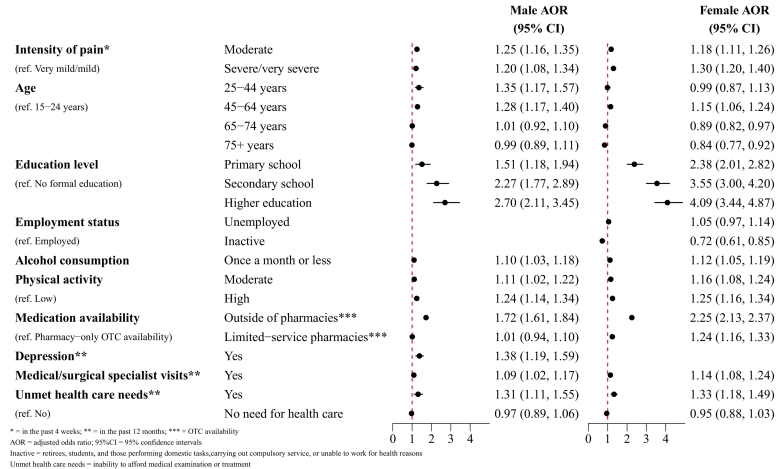
Multivariable analysis of self-medication in non-institutionalized residents aged 15 and over experiencing pain in the European Union. European Health Interview Survey Wave 3 (2018–2020).

The results of the multivariable analysis are displayed graphically in Fig. 1 and in table-form in Table 4. The results demonstrate statistically significant differences in self-medication between pain intensities. While self-medication among females is incrementally affected by increasing pain (moderate AOR = 1.18;95%CI = 1.11,1.26, severe/very severe AOR = 1.30;95%CI = 1.20,1.40), self-medication among males is more affected by moderate pain than severe/very severe pain (moderate AOR = 1.25;95%CI = 1.16,1.35, severe/very severe AOR = 1.20;95%CI = 1.08,1.34). Both education level (higher education vs. no formal education: male AOR = 2.70;95%CI = 2.11,3.45, female AOR = 4.09;95%CI = 3.44,4.87) and physical activity (high vs. low: male AOR = 1.24;95%CI = 1.14,1.34, female AOR = 1.25;95%CI = 1.16,1.34) are also associated with self-medication. In men, a statistically significant association was found between self-medication and depression (AOR = 1.38;95%CI = 1.19,1.59). Other statistically significant variables for self-medication are the availability of medications (non-pharmacy vs. pharmacy-only availability: male AOR = 1.72;95%CI = 1.61,1.84, female AOR = 2.25;95%CI = 2.13,2.37) and unmet health care needs due to economic barriers (male AOR = 1.31;95%CI = 1.20,1.45, female AOR = 1.33;95%CI = 1.18,1.49).

**Table 4:** Multivariable analysis of self-medication in non-institutionalized residents aged 15 and over experiencing pain during a four-week window in the European Union. European Health Interview Survey Wave 3 (2018–2020).

		Male	Female	Both Sexes
Variable	Category	AOR (95%CI)	AOR (95%CI)	AOR (95%CI)
Intensity of pain in the past four weeks	Very mild/mild	1	1	1
	Moderate	1.25 (1.16, 1.35)	1.18 (1.11, 1.26)	1.21 (1.15, 1.27)
	Severe/very severe	1.20 (1.08, 1.34)	1.30 (1.20, 1.40)	1.26 (1.18, 1.34)
				
Age	15–24 years	1	1	1
	25–44 years	1.35 (1.17, 1.57)	0.99 (0.87, 1.13)	1.14 (1.03, 1.27)
	45–64 years	1.28 (1.17, 1.40)	1.15 (1.06, 1.24)	1.19 (1.13, 1.27)
	65–74 years	1.01 (0.92, 1.10)	0.89 (0.82, 0.97)	0.96 (0.90, 1.03)
	75+ years	0.99 (0.89, 1.11)	0.84 (0.77, 0.92)	0.92 (0.85, 0.99)
				
Education level	No formal education	1	1	1
	Primary school	1.51 (1.18, 1.94)	2.38 (2.01, 2.82)	2.14 (1.85, 2.47)
	Secondary school	2.27 (1.77, 2.89)	3.55 (3.00, 4.20)	3.18 (2.76, 3.66)
	Higher education	2.70 (2.11, 3.45)	4.09 (3.44, 4.87)	3.72 (3.22, 4.30)
				
Employment status	Employed	NS	1	1
	Unemployed	NS	1.05 (0.97, 1.14)	1.06 (1.00, 1.13)
	Inactive	NS	0.72 (0.61, 0.85)	0.80 (0.70, 0.91)
				
Alcohol consumption	More than once a month	1	1	1
	Once a month or less	1.10 (1.03, 1.18)	1.12 (1.05, 1.19)	1.10 (1.05, 1.15)
			
Physical activity levels	Low	1	1	1
	Moderate	1.11 (1.02, 1.22)	1.16 (1.08, 1.24)	1.14 (1.08, 1.21)
	High	1.24 (1.14, 1.34)	1.25 (1.16, 1.34)	1.25 (1.19, 1.32)
				
Medication availability cluster	Pharmacy-only OTC availability	1	1	1
	OTC availability outside of pharmacies	1.72 (1.61, 1.84)	2.25 (2.13, 2.37)	2.02 (1.94, 2.11)
	OTC availability in limited-service pharmacies	1.01 (0.94, 1.10)	1.24 (1.16, 1.33)	1.15 (1.09, 1.21)
				
Depression⁎⁎	Yes	1.38 (1.19, 1.59)	NS	1.18 (1.08, 1.28)
	No	1	NS	1
				
Visit to a medical or surgical specialist⁎⁎	Yes	1.09 (1.02, 1.17)	1.14 (1.08, 1.21)	1.12 (1.07, 1.17)
	No	1	1	1
				
Unmet need for health care due to inability to afford medical examination or treatment⁎⁎	Yes	1.31 (1.11, 1.55)	1.33 (1.18, 1.49)	1.32 (1.20, 1.45)
	No	1	1	1
	No need for health care	0.97 (0.89, 1.06)	0.95 (0.88, 1.03)	0.96 (0.90, 1.02)

⁎⁎ = in the past 12 months; AOR = adjusted odds ratio; 95%CI = 95% confidence intervals; NS = not significant; Inactive = retirees, students, and those performing domestic tasks, carrying out compulsory service, or unable to work for health.

## 4 Discussion

From a European perspective, this article identifies the factors associated with self-medication among patients suffering from pain. Our results indicate that 42.7% of the European Union population that experiences pain self-medicates, with women showing a higher prevalence of non-prescribed consumption than men, in line with the results obtained in other studies (Dale et al., 2015; Knopf et al., 2017). This finding aligns with data from the Nord-Trøndelag Health Study (HUNT3, 2006–08) which showed a higher prevalence (64.1%) of OTC analgesic use among individuals with chronic pain versus those without chronic pain (39.2%), as well as consistently higher rates in women—another finding in line with our results (Dale et al., 2015). The prevalence found in this study is also lower than that reported by Breivik et al. (2006), who found that 47% of respondents experiencing chronic pain across 15 European countries self-medicated with analgesics.

The prevalence of self-medication among patients with pain varies between countries, with lower frequencies in countries such as Spain and Italy and higher prevalence values in countries like Finland and Cyprus. Self-medication among people with pain is more common in Northern and Eastern European countries than in Western and Southern Europe, in line with other research on the European Union population that also highlight these differences (Yeamans et al., 2024). The variations in self-medication between countries illustrate how cultural factors such as beliefs and ethnicity, socioeconomic factors such as income and education, and health-system factors related to access and quality all shape health decisions. These factors create a context in which people choose self-medication based not only on what is medically ideal, but also on what is culturally acceptable, economically feasible, and accessible within the health-care system. The study by Vowles et al., conducted through a survey in a European context, including Belgium, France, Germany, Great Britain, Italy, Poland, Russia, and Spain, which examined cultural factors such as beliefs and attitudes related to analgesic use (Vowles et al., 2014), shows that people living in Russia and Poland express greater concern about the consumption of analgesics. They also note that the use of these medications had little correlation with their knowledge about them, in contrast to the perception of analgesics in southern countries such as Spain and Italy.

With respect to the health-care system, high rates of medication use in a population could indicate strong health systems, suggesting potentially higher adherence rates due to better access to medicines and more efficient health-care processes. A recent study conducted in 39 countries across Europe and Israel to address medication non-adherence in health care found differences in medication-use percentages ranging from 23% in Romania to 71% in Norway (Ágh et al., 2024),suggesting different patterns of health-service use that could also influence levels of medication adherence and, consequently, increase the likelihood of resorting to self-medication.

Related to the characteristics of each country's health system is the association found in this study between the availability of over-the-counter medications and self-medication in people suffering from pain. Our results show that the likelihood of self-medication is more than double in countries where OTC medications are purchasable outside of pharmacies versus countries where OTC medications are sold exclusively in pharmacies. This not only supports the idea that increasing points of purchase for OTC drugs increases their use (Oleszkiewicz et al., 2021), but the strength of the association also highlights the substantial impact that pharmaceutical policy decisions have on patient behavior and the need to improve knowledge about the appropriate use of OTC medications. However, other researchers using EHIS data with different clusters and methodologies did not find a link between market regulation and OTC consumption (Tavares, 2025), indicating the need for further research into this relationship.

Regarding pain intensity, our data show that increased pain levels are significantly associated with a greater likelihood of self-medication, in line with other research (Zerriouh et al., 2024). A study by Perrot et al. found similar results when analyzing chronic low back pain using data from the National Health and Wellness Survey in Germany, France, the UK, Italy, and Spain, with their findings showing a higher frequency of self-medication as pain intensity increased (2019).

Notably, we found significant differences in self-medication for men and women in response to pain severity, with possible implications for how we understand pain perception and management. Severe pain leads the women in the study to a higher likelihood of self-medicating. Parra-Fernández et al. (2020), using a questionnaire on menstrual pain and self-care, found a higher frequency of self-medicated treatment as pain levels increased among the participating women. Men, however, are most likely to self-medicate with moderate pain. This difference in self-medication between men and women could be explained by a different perception of pain and a greater sensitivity to pain among women, as documented in various studies (Coppola et al., 2025; Bartley and Fillingim, 2013; Keogh, 2025; Zimmer et al., 2022), in addition to the scientific evidence showing higher consumption of analgesics, both prescribed and self-medicated, among the female population (Dale et al., 2015; Sánchez-Sánchez et al., 2021; Chilet-Rosell et al., 2013; Ruiz-Cantero et al., 2020).

One additional characteristic that differs in the pattern of self-medication between men and women with pain is age. The results indicate that men experiencing pain are most likely to self-medicate between 25 and 44 years of age, whereas women are most likely to do so between 45 and 64 years old. Previous research has found that self-medication typically decreases with age, often attributable to rising comorbidities and worse health perception that leads to greater contact with medical services and the use of subsequent prescription medicines (Knopf et al., 2017; Heidemann et al., 2021). The later peak observed among women in our study could reflect factors affecting women in midlife, such as the physiological changes of menopause.

There is also evidence that pain intensity is associated with mental health factors, particularly depression, as shown in a study of 15 European countries which found that 21% of respondents with chronic pain were diagnosed with depression as a direct consequence of their pain (Breivik et al., 2006). A possible and complex relationship between the use of OTC analgesics and anxiety and depression has been identified, particularly in certain population groups (Roalsø et al., 2024). Several studies indicate that the coexistence of pain, anxiety, and depression is more frequent in women, who may resort to self-medication with OTC analgesics to mitigate these symptoms (Jonassen et al., 2021; Koushede et al., 2010). The results of our study, by contrast, show a statistically significant association between depression and self-medication in male patients experiencing pain, with no statistically significant association observed in women.

In line with other studies, our findings indicate a significant association between self-medication and higher educational level among individuals with pain. Men and women with higher education were more than 2.5 times and four times as likely, respectively, to self-medicate compared to those without formal education. This observation agrees with the findings of other studies showing an association between the use of OTC medicines and a high educational level (Carrasco-Garrido et al., 2014). Although a higher educational level could be associated with greater health literacy and knowledge about medications, it could also reduce the perceived risk of self-medication, creating a scenario where inappropriate use of these medications may generate avoidable health care costs due to irresponsible self-medication (Brandão et al., 2020; Mallah et al., 2022; Vong et al., 2022).

The analysis of lifestyle factors showed that individuals experiencing pain in our study who consume alcohol had a higher likelihood of resorting to self-medication than those who do not consume alcohol. A recent study carried out by Mizuno et al. (2024) using data from a national survey conducted in Japan in 2023 shows that overdose of OTC medications was associated with excessive alcohol consumption, with medications mainly obtained from pharmacies. By contrast, the results of the study by Dale et al. (2015) demonstrated alcohol consumption 2 to 7 times per week reduced the risk of daily use of OTC analgesics in subjects with chronic pain compared with non-consumers of alcohol. Undoubtedly, understanding the characteristics of alcohol consumption habits among individuals who use OTC medications is important for reducing health risks.

Finally, this study results show an association between self-medication among people with pain and unmet needs for medical care and treatment due to economic difficulties, in line with results from the general population (Yeamans et al., 2024). This result underscores the need to improve access to healthcare, ensuring that populations in vulnerable situations, where access to healthcare products and medications is not always guaranteed, do not have self-medication as their only available resource.

This study is subject to a series of limitations. First, due to the cross-sectional nature of the data, causality cannot be determined. Second, the EHIS does not collect drug class data, which prevents the exploration of variations in self-medication across different types of medicines. Another limitation is the absence of data on the origin, frequency, duration, and typology of the pain. Additionally, social desirability bias could have led to underreporting on self-medication and pain. For example, individuals interviewed face-to-face or over the phone may have felt pressure to underreport self-medication and pain versus those who filled out surveys. These cross-country variations in data collection methods and sampling design may reduce comparability. Similarly, because data was collected non-simultaneously across years and seasons, seasonal or annual fluctuations could have influenced results. Moreover, three countries (Germany, Spain, and Malta) collected data after the onset of the COVID-19 pandemic, potentially altering outcomes (European Commission. Statistical Office of the European Union., 2022). The data is also susceptible to recall bias, and the non-response rates, ranging from 12% to 78% across countries (European Commission. Statistical Office of the European Union., 2022), may also have impacted results, as non-participants could differ in their self-medication behaviors, though the direction of this bias is unclear.

Nonetheless, the EHIS-3 quality report explains the rigorous validation, calibration, and non- response adjustment procedures to mitigate potential sources of both sampling and non-sampling errors in the data. These processes resulted in a highly harmonized dataset, ensuring a high level of comparability across European Union member states (European Commission. Statistical Office of the European Union., 2022). Combined with the robust weighted sample size of 149,349, these limitations should not compromise the relevance of the findings.

## 5 Conclusions

The prevalence of self-medication among people with pain in the European Union is 42.7%, with higher values in women, variation between countries, and a higher prevalence than in the general population. Likewise, self-medication is significantly associated with a higher educational level. Self-medication is also influenced by pain intensity, with differences for men and women based on pain level. A key finding is that the availability of OTC medications outside pharmacies is significantly associated with self-medication, as are economic barriers to healthcare access.

It is necessary to identify an appropriate context for self-medication in people with pain, incorporating training on the correct use of medications into general health education.

The following are the supplementary data related to this article.

Supplementary Table S1 Prevalence by country of self-medication in non-institutionalized residents aged 15 and over experiencing pain in the European Union. European Health Interview Survey Wave 3 (2018-2020).

Supplementary Table S2 Prevalence of self-medication in non-institutionalized residents of both sexes aged 15 and over experiencing pain in the European Union. European Health Interview Survey Wave 3 (2018-2020).

## Code availability

The code for data processing is available upon request to the authors.

## CRediT authorship contribution statement

**Spencer Yeamans:** Writing – original draft, Visualization, Project administration, Data curation, Conceptualization. **Ángel Gil-De-Miguel:** Writing – review & editing, Supervision, Project administration, Conceptualization. **Valentín Hernández-Barrera:** Writing – review & editing, Formal analysis, Data curation, Conceptualization. **Pilar Carrasco-Garrido:** Writing – review & editing, Supervision, Project administration, Conceptualization. All authors read and approved the final manuscript.

## Funding

Spencer Yeamans is the recipient of funding from a Universidad Rey Juan Carlos predoctoral program (reference code: PREDOC20–107). The funding source was not involved in this study.

## Declaration of competing interest

The authors declare that they have no known competing financial interests or personal relationships that could have appeared to influence the work reported in this paper.

## Data Availability

Data requests must be made to Eurostat. Instructions for requesting data access can be found at https://ec.europa.eu/eurostat/web/microdata/european-health-interview-survey.
