# Supplementary material for: Self-medication and pain in the European Union: Gender differences and associated factors
Source: Prev Med Rep. 2026 May 4;66:103488. doi: 10.1016/j.pmedr.2026.103488 (PMC13156551; doi:10.1016/j.pmedr.2026.103488)
Supplement: Supplementary Table S1 — Prevalence by country of self-medication in non-institutionalized residents aged 15 and over experiencing pain in the European Union. European Health Interview Survey Wave 3 (2018-2020). [file mmc1.docx]

**Supplementary Table 1. Prevalence by country of self-medication in non-institutionalized residents aged 15 and over experiencing pain in the European Union.**

**European Health Interview Survey Wave 3 (2018-2020)**

|  | **Very Mild/Mild** | | | | | **Moderate** | | | | | **Severe/Very Severe** | | | | |
| --- | --- | --- | --- | --- | --- | --- | --- | --- | --- | --- | --- | --- | --- | --- | --- |
|  | **Male** | | **Female** | | **OR** | **Male** | | **Female** | | **OR** | **Male** | | **Female** | | **OR** |
|  | **N** | **% (95%CI)** | **N** | **% (95%CI)** |  | **N** | **% (95%CI)** | **N** | **% (95%CI)** |  | **N** | **% (95%CI)** | **N** | **% (95%CI)** |  |
| **Austria** | 710 | 37.4 (37.3, 37.5) | 1085 | 50.1 (50.0, 50.2) | 1.68 (1.45, 1.95) | 371 | 36.6 (36.5, 36.8) | 837 | 55.2 (55.1, 55.4) | 2.13 (1.75, 2.60) | 255 | 33.4 (33.2, 33.5) | 657 | 53.0 (52.8, 53.1) | 2.25 (1.79, 2.83) |
| **Belgium** | 374 | 31.1 (31.0, 31.2) | 541 | 37.1 (37.1, 37.2) | 1.31 (1.05, 1.63) | 217 | 34.4 (34.3, 34.5) | 368 | 40.8 (40.7, 40.9) | 1.31 (0.99, 1.75) | 98 | 29.1 (28.9, 29.2) | 248 | 40.2 (40.1, 40.3) | 1.64 (1.08, 2.48) |
| **Bulgaria** | 389 | 43.0 (42.9, 43.2) | 686 | 57.8 (57.7, 57.9) | 1.81 (1.51, 2.18) | 165 | 50.5 (50.3, 50.7) | 319 | 57.6 (57.4, 57.8) | 1.33 (0.99, 1.78) | 68 | 56.0 (55.7, 56.3) | 137 | 61.3 (61.1, 61.6) | 1.25 (0.78, 1.99) |
| **Cyprus** | 341 | 63.8 (63.4, 64.2) | 496 | 71.4 (71.1, 71.7) | 1.42 (1.09, 1.85) | 135 | 67.1 (66.5, 67.8) | 240 | 79.0 (78.5, 79.5) | 1.84 (1.19, 2.86) | 147 | 77.5 (77.0, 78.1) | 252 | 76.2 (75.7, 76.6) | 0.93 (0.57, 1.51) |
| **Czechia** | 603 | 55.3 (55.3, 55.4) | 1040 | 68.7 (68.7, 68.8) | 1.77 (1.48, 2.12) | 277 | 63.7 (63.6, 63.9) | 483 | 69.4 (69.3, 69.5) | 1.29 (0.97, 1.71) | 92 | 59.5 (59.3, 59.8) | 208 | 68.7 (68.5, 68.9) | 1.49 (0.95, 2.35) |
| **Germany** | 1313 | 35.6 (35.6, 35.6) | 2013 | 47.9 (47.8, 47.9) | 1.66 (1.47, 1.87) | 479 | 40.1 (40.0, 40.2) | 1125 | 51.4 (51.4, 51.5) | 1.58 (1.31, 1.91) | 316 | 36.2 (36.1, 36.2) | 722 | 48.4 (48.4, 48.5) | 1.66 (1.33, 2.07) |
| **Denmark** | 576 | 47.4 (47.3, 47.5) | 1110 | 66.5 (66.4, 66.6) | 2.20 (1.87, 2.59) | 213 | 55.3 (55.1, 55.4) | 417 | 67.0 (66.9, 67.2) | 1.65 (1.24, 2.18) | 117 | 58.6 (58.4, 58.8) | 235 | 64.8 (64.6, 65.0) | 1.30 (0.89, 1.90) |
| **Estonia** | 240 | 43.9 (43.6, 44.2) | 432 | 59.6 (59.4, 59.9) | 1.89 (1.49, 2.40) | 214 | 54.0 (53.7, 54.3) | 476 | 69.6 (69.3, 69.8) | 1.95 (1.48, 2.56) | 135 | 62.5 (62.1, 62.9) | 371 | 79.6 (79.3, 79.8) | 2.34 (1.59, 3.42) |
| **Greece** | 177 | 23.9 (23.8, 24.0) | 300 | 28.5 (28.5, 28.6) | 1.27 (0.99, 1.64) | 91 | 27.3 (27.1, 27.4) | 190 | 31.7 (31.6, 31.8) | 1.24 (0.87, 1.76) | 65 | 34.5 (34.3, 34.7) | 137 | 33.8 (33.6, 33.9) | 0.97 (0.62, 1.50) |
| **Spain** | 399 | 21.4 (21.4, 21.4) | 613 | 24.5 (24.5, 24.6) | 1.19 (1.00, 1.42) | 204 | 21.0 (20.9, 21.0) | 386 | 21.7 (21.7, 21.8) | 1.05 (0.83, 1.33) | 78 | 20.2 (20.1, 20.3) | 182 | 19.3 (19.3, 19.4) | 0.95 (0.66, 1.35) |
| **Finland** | 639 | 66.0 (65.9, 66.1) | 1115 | 77.9 (77.8, 77.9) | 1.80 (1.48, 2.20) | 311 | 72.4 (72.3, 72.6) | 629 | 85.1 (85.0, 85.2) | 2.17 (1.58, 2.97) | 106 | 68.0 (67.8, 68.2) | 245 | 81.9 (81.8, 82.1) | 2.13 (1.31, 3.46) |
| **Croatia** | 257 | 32.6 (32.5, 32.7) | 478 | 46.2 (46.1, 46.3) | 1.78 (1.38, 2.89) | 124 | 39.9 (39.7, 40.1) | 275 | 49.1 (48.9, 49.3) | 1.45 (1.02, 2.07) | 83 | 47.2 (46.9, 47.5) | 206 | 54.6 (54.4, 54.8) | 1.34 (0.84, 2.14) |
| **Hungary** | 339 | 55.6 (55.5, 55.7) | 496 | 66.5 (66.4, 66.6) | 1.58 (1.26, 1.99) | 186 | 62.3 (62.1, 62.4) | 349 | 69.0 (68.9, 69.2) | 1.35 (0.99, 1.85) | 105 | 62.8 (62.6, 63.0) | 220 | 69.6 (69.5, 69.7) | 1.35 (0.89, 2.06) |
| **Ireland** | 281 | 34.2 (34.0, 34.3) | 404 | 42.3 (42.1, 42.4) | 1.41 (1.11, 1.79) | 120 | 35.0 (34.8, 35.2) | 236 | 41.5 (41.3, 41.7) | 1.32 (0.94, 1.85) | 79 | 30.1 (29.9, 30.4) | 166 | 44.1 (43.9, 44.4) | 1.83 (1.23, 2.72) |
| **Italy** | 762 | 22.5 (22.5, 22.6) | 998 | 24.7 (24.6, 24.8) | 1.13 (0.98, 1.29) | 566 | 21.0 (21.0, 21.1) | 1047 | 25.8 (25.7, 25.9) | 1.30 (1.13, 1.51) | 337 | 22.5 (22.4, 22.6) | 777 | 27.3 (27.2, 27.4) | 1.29 (1.08, 1.55) |
| **Lithuania** | 278 | 52.9 (52.7, 53.0) | 547 | 70.5 (70.3, 70.6) | 2.13 (1.68, 2.70) | 244 | 64.9 (64.7, 65.1) | 566 | 79.1 (78.9, 79.2) | 2.04 (1.53, 2.73) | 88 | 63.2 (62.9, 63.6) | 309 | 79.4 (79.2, 79.6) | 2.24 (1.45, 3.48) |
| **Luxembourg** | 284 | 34.5 (34.2, 34.8) | 458 | 45.7 (45.4, 46.0) | 1.60 (1.31, 1.95) | 116 | 34.3 (33.8, 34.7) | 221 | 47.1 (46.6, 47.5) | 1.71 (1.26, 2.30) | 62 | 38.9 (38.2, 39.7) | 108 | 43.0 (42.4, 43.6) | 1.18 (0.78, 1.79) |
| **Latvia** | 353 | 49.5 (49.2, 49.7) | 781 | 64.8 (64.6, 64.9) | 1.88 (1.53, 2.30) | 92 | 53.1 (52.6, 53.5) | 254 | 67.0 (66.7, 67.3) | 1.80 (1.19, 2.71) | 48 | 52.5 (51.9, 53.1) | 136 | 60.3 (59.9, 60.8) | 1.38 (0.79, 2.38) |
| **Malta** | 201 | 34.1 (33.8, 34.5) | 357 | 48.5 (48.1, 48.9) | 1.81 (1.43, 2.30) | 45 | 44.6 (43.7, 45.6) | 123 | 57.3 (56.6, 58.0) | 1.66 (1.01, 2.74) | 25 | 35.6 (34.4, 36.8) | 73 | 46.0 (45.1, 46.8) | 1.53 (0.83, 2.84) |
| **Netherlands** | 550 | 37.4 (37.3, 37.4) | 861 | 51.9 (51.8, 51.9) | 1.81 (1.55, 2.09) | 200 | 46.2 (46.1, 46.3) | 365 | 57.9 (57.9, 58.0) | 1.60 (1.24, 2.07) | 144 | 45.7 (45.6, 45.8) | 327 | 58.6 (58.5, 58.7) | 1.68 (1.26, 2.24) |
| **Poland** | 754 | 43.6 (43.5, 43.6) | 1541 | 59.9 (59.8, 59.9) | 1.93 (1.65, 2.26) | 623 | 48.9 (48.9, 49.0) | 1466 | 63.6 (63.5, 63.6) | 1.82 (1.53, 2.17) | 273 | 49.6 (49.5, 49.7) | 819 | 68.4 (68.3, 68.5) | 2.19 (1.69, 2.84) |
| **Portugal** | 386 | 24.1 (24.0, 24.1) | 637 | 27.3 (27.3, 27.4) | 1.19 (0.94, 1.50) | 229 | 28.8 (28.7, 28.9) | 504 | 30.0 (29.9, 30.1) | 1.06 (0.79, 1.42) | 162 | 24.8 (24.7, 25.0) | 441 | 26.0 (25.9, 26.1) | 1.06 (0.77, 1.48) |
| **Romania** | 482 | 19.0 (18.9, 19.0) | 901 | 31.7 (31.6, 31.7) | 1.97 (1.69, 2.31) | 378 | 29.3 (29.2, 29.4) | 588 | 34.5 (34.4, 34.6) | 1.27 (1.06, 1.53) | 101 | 32.8 (32.6, 33.0) | 185 | 36.0 (35.8, 36.1) | 1.15 (0.81, 1.62) |
| **Sweden** | 865 | 35.4 (35.4, 35.5) | 1079 | 47.6 (47.5, 47.7) | 1.65 (1.47, 1.87) | 325 | 42.1 (42.0, 42.3) | 514 | 48.7 (48.6, 48.8) | 1.30 (1.07, 1.59) | 113 | 40.2 (40.0, 40.4) | 257 | 54.3 (54.2, 54.5) | 1.77 (1.30, 2.42) |
| **Slovenia** | 505 | 32.4 (32.2, 32.5) | 866 | 45.0 (44.8, 45.2) | 1.71 (1.47, 1.98) | 299 | 35.2 (34.9, 35.4) | 635 | 48.5 (48.3, 48.7) | 1.73 (1.44, 2.09) | 117 | 37.7 (37.4, 38.1) | 294 | 47.9 (47.6, 48.2) | 1.52 (1.13, 2.04) |
| **Slovakia** | 268 | 54.0 (53.8, 54.1) | 521 | 66.4 (66.3, 66.6) | 1.69 (1.30, 2.17) | 173 | 57.3 (57.1, 57.5) | 426 | 67.2 (67.0, 67.3) | 1.52 (1.13, 2.06) | 127 | 65.9 (65.6, 66.1) | 362 | 77.9 (77.7, 78.0) | 1.82 (1.22, 2.72) |
| **Total** | 12326 | 35.5 (35.5, 35.5) | 20356 | 46.4 (46.3, 46.4) | 1.57 (1.50, 1.64) | 6397 | 39.4 (39.3, 39.4) | 13039 | 47.8 (47.8, 47.8) | 1.41 (1.32, 1.50) | 3341 | 38.4 (38.4, 38.4) | 8074 | 47.4 (47.4, 47.5) | 1.45 (1.33, 1.57) |

95%CI = 95% confidence intervals; OR = odds ratio
