# Supplementary material for: Self-medication and pain in the European Union: Gender differences and associated factors
Source: Prev Med Rep. 2026 May 4;66:103488. doi: 10.1016/j.pmedr.2026.103488 (PMC13156551; doi:10.1016/j.pmedr.2026.103488)
Supplement: Supplementary Table S2 — Prevalence of self-medication in non-institutionalized residents of both sexes aged 15 and over experiencing pain in the European Union. European Health Interview Survey Wave 3 (2018-2020). [file mmc2.docx]

**Supplementary Table 2. Prevalence of self-medication in non-institutionalized residents of both sexes aged 15 and over experiencing pain in the European Union. European Health Interview Survey Wave 3 (2018-2020)**

|  |  | **Very Mild/Mild Pain** | | **Moderate Pain** | | **Severe/Very Severe Pain** | |
| --- | --- | --- | --- | --- | --- | --- | --- |
|  |  | **N** | **% (95%CI)** | **N** | **% (95%CI)** | **N** | **% (95%CI)** |
| **Age** | 15-24 years | 2545 | 42.4 (42.4-42.5) | 869 | 46.4 (46.4-46.5) | 425 | 47.3 (47.2-47.4) |
|  | 25-44 years | 8533 | 46.8 (46.7-46.8) | 3736 | 51.6 (51.6-51.6) | 2013 | 53.0 (52.9-53.0) |
|  | 45-64 years | 12001 | 40.7 (40.6-40.7) | 7336 | 45.1 (45.1-45.1) | 4313 | 44.6 (44.6-44.6) |
|  | 65-74 years | 5830 | 38.2 (38.1-38.2) | 3960 | 41.3 (41.2-41.3) | 2146 | 42.1 (42.0-42.1) |
|  | 75+ years | 3773 | 33.4 (33.3-33.4) | 3535 | 37.5 (37.5-37.5) | 2518 | 37.0 (37.0-37.1) |
|  |  | 29498 | 41.4 (41.4-41.5) | 17594 | 44.5 (44.5-44.5) | 10188 | 44.3 (44.3-44.3) |
| **Nationality*** | Native-born | 1338 | 44.8 (44.8-44.9) | 722 | 45.8 (45.7-45.9) | 442 | 44.9 (44.8-45.0) |
|  | Born in another EU state | 1769 | 37.9 (37.8-37.9) | 1090 | 42.6 (42.5-42.6) | 757 | 41.4 (41.3-41.4) |
|  | Born in non-EU country | 250 | 14.2 (14.2-14.3) | 300 | 17.5 (17.5-17.6) | 297 | 20.4 (20.3-20.5) |
|  |  | 6476 | 31.9 (31.9-31.9) | 4833 | 33.9 (33.9-33.9) | 3396 | 35.3 (35.3-35.3) |
| **Education level** | No formal education | 13682 | 42.5 (42.4-42.5) | 8607 | 48.6 (48.6-48.7) | 4950 | 48.9 (48.9-48.9) |
|  | Primary school | 12047 | 48.5 (48.5-48.6) | 5596 | 52.6 (52.5-52.6) | 2705 | 53.3 (53.2-53.3) |
|  | Secondary school | 16873 | 44.2 (44.1-44.2) | 8057 | 49.6 (49.6-49.6) | 3945 | 49.7 (49.7-49.7) |
|  | Higher education | 1077 | 34.6 (34.5-34.7) | 706 | 35.5 (35.4-35.6) | 542 | 39.9 (39.8-39.9) |
|  |  | 14538 | 38.5 (38.5-38.5) | 10564 | 41.1 (41.1-41.1) | 6862 | 41.3 (41.3-41.3) |
| **Employment status** | Employed | 6386 | 39.8 (39.7-39.8) | 3910 | 44.9 (44.9-44.9) | 2512 | 44.7 (44.6-44.7) |
|  | Unemployed | 26098 | 41.9 (41.9-41.9) | 15379 | 44.4 (44.4-44.4) | 8819 | 44.1 (44.1-44.2) |
|  | Inactive | 10616 | 41.5 (41.5-41.5) | 5089 | 46.5 (46.4-46.5) | 2549 | 46.4 (46.4-46.4) |
|  |  | 18343 | 41.2 (41.2-41.2) | 11664 | 44.5 (44.5-44.5) | 7325 | 44.8 (44.7-44.8) |
| **Smoking** | Yes | 16298 | 38.6 (38.6-38.6) | 10891 | 40.7 (40.6-40.7) | 7115 | 41.2 (41.2-41.3) |
|  | No | 7171 | 42.4 (42.4-42.4) | 3782 | 47.0 (47.0-47.0) | 1861 | 46.7 (46.6-46.7) |
|  |  | 9126 | 45.8 (45.7-45.8) | 4691 | 51.8 (51.8-51.8) | 2400 | 50.8 (50.8-50.9) |
| **Alcohol consumption** | More than once a month | 10595 | 37.6 (37.6-37.6) | 6344 | 38.3 (38.2-38.3) | 3968 | 39.5 (39.5-39.6) |
|  | Once a month or less | 14545 | 46.5 (46.4-46.5) | 8906 | 49.6 (49.6-49.6) | 5026 | 49.9 (49.9-49.9) |
|  |  | 7542 | 38.2 (38.2-38.2) | 4186 | 42.7 (42.7-42.8) | 2421 | 40.9 (40.8-40.9) |
| **Physical activity** | Low | 21578 | 42.2 (42.2-42.2) | 7572 | 47.1 (47.1-47.1) | 2952 | 47.1 (47.0-47.1) |
|  | Moderate | 9502 | 40.1 (40.1-40.1) | 8820 | 43.7 (43.6-43.7) | 4136 | 45.3 (45.3-45.4) |
|  | High | 1515 | 35.8 (35.8-35.9) | 2976 | 39.7 (39.7-39.8) | 4290 | 41.1 (41.1-41.2) |
|  |  | 17602 | 41.8 (41.8-41.8) | 14384 | 44.2 (44.2-44.2) | 9488 | 44.2 (44.2-44.3) |
| **Medication availability cluster** | Pharmacy-only OTC availability | 14924 | 40.9 (40.9-40.9) | 4932 | 45.1 (45.0-45.1) | 1873 | 43.9 (43.9-44.0) |
|  | OTC availability outside of pharmacies | 2239 | 46.6 (46.6-46.7) | 2369 | 46.8 (46.7-46.8) | 2369 | 44.7 (44.6-44.7) |
|  | OTC availability in limited-service pharmacies | 30052 | 40.9 (40.9-40.9) | 16763 | 44.0 (44.0-44.0) | 8916 | 43.9 (43.9-43.9) |
|  |  | 25903 | 41.8 (41.8-41.8) | 16930 | 44.2 (44.2-44.2) | 10402 | 43.9 (43.9-44.0) |
| **Self-perceived health** | Very good/good | 6718 | 39.9 (39.8-39.9) | 2471 | 46.7 (46.7-46.8) | 983 | 47.1 (47.1-47.2) |
|  | Fair | 18166 | 43.3 (43.3-43.3) | 12870 | 45.3 (45.3-45.3) | 8573 | 44.8 (44.7-44.8) |
|  | Bad/very bad | 14394 | 39.2 (39.2-39.2) | 6489 | 43.0 (43.0-43.0) | 2792 | 42.6 (42.6-42.6) |
|  |  | 1523 | 49.6 (49.5-49.6) | 1552 | 47.7 (47.7-47.8) | 1484 | 50.6 (50.5-50.6) |
| **Long-standing health problem** | Yes | 22918 | 41.5 (41.5-41.5) | 14225 | 44.2 (44.1-44.2) | 8288 | 43.6 (43.5-43.6) |
|  | No | 7172 | 40.8 (40.8-40.8) | 2962 | 46.2 (46.2-46.3) | 1212 | 44.9 (44.9-45.0) |
|  |  | 2545 | 42.4 (42.4-42.5) | 869 | 46.4 (46.4-46.5) | 425 | 47.3 (47.2-47.4) |
| **Depression**** | Yes | 8533 | 46.8 (46.7-46.8) | 3736 | 51.6 (51.6-51.6) | 2013 | 53.0 (52.9-53.0) |
|  | No | 12001 | 40.7 (40.6-40.7) | 7336 | 45.1 (45.1-45.1) | 4313 | 44.6 (44.6-44.6) |
|  |  | 5830 | 38.2 (38.1-38.2) | 3960 | 41.3 (41.2-41.3) | 2146 | 42.1 (42.0-42.1) |
| **Visit to a general practitioner or family doctor**** | Yes | 3773 | 33.4 (33.3-33.4) | 3535 | 37.5 (37.5-37.5) | 2518 | 37.0 (37.0-37.1) |
|  | No | 29498 | 41.4 (41.4-41.5) | 17594 | 44.5 (44.5-44.5) | 10188 | 44.3 (44.3-44.3) |
|  |  | 1338 | 44.8 (44.8-44.9) | 722 | 45.8 (45.7-45.9) | 442 | 44.9 (44.8-45.0) |
| **Visit to a medical or surgical specialist**** | Yes | 1769 | 37.9 (37.8-37.9) | 1090 | 42.6 (42.5-42.6) | 757 | 41.4 (41.3-41.4) |
|  | No | 250 | 14.2 (14.2-14.3) | 300 | 17.5 (17.5-17.6) | 297 | 20.4 (20.3-20.5) |
|  |  | 6476 | 31.9 (31.9-31.9) | 4833 | 33.9 (33.9-33.9) | 3396 | 35.3 (35.3-35.3) |
| **Unmet need for health care due to inability to afford medical examination or treatment**** | Yes | 13682 | 42.5 (42.4-42.5) | 8607 | 48.6 (48.6-48.7) | 4950 | 48.9 (48.9-48.9) |
|  | No | 12047 | 48.5 (48.5-48.6) | 5596 | 52.6 (52.5-52.6) | 2705 | 53.3 (53.2-53.3) |
|  | No need for health care | 16873 | 44.2 (44.1-44.2) | 8057 | 49.6 (49.6-49.6) | 3945 | 49.7 (49.7-49.7) |

* = does not include data from Malta; ** = in the past 12 months; 95%CI = 95% confidence intervals; EU = European Union; Inactive = retirees, students, and those performing domestic tasks, carrying out compulsory service, or unable to work for health reasons
